# Mitochondrial Transfer via Tunneling Nanotubes is an Important Mechanism by Which Mesenchymal Stem Cells Enhance Macrophage Phagocytosis in the In Vitro and In Vivo Models of ARDS

**DOI:** 10.1002/stem.2372

**Published:** 2016-04-29

**Authors:** Megan V. Jackson, Thomas J. Morrison, Declan F. Doherty, Daniel F. McAuley, Michael A. Matthay, Adrien Kissenpfennig, Cecilia M. O'Kane, Anna D. Krasnodembskaya

**Affiliations:** ^1^ Centre for Experimental Medicine, School of Medicine Dentistry & Biomedical SciencesQueen's University BelfastNorthern IrelandUK; ^2^ Department of Anaesthesiology & MedicineUniversity of California San FranciscoSan FranciscoCaliforniaUSA

**Keywords:** Mesenchymal stem cells, Macrophages, Mitochondrial transfer, ARDS, Phagocytosis

## Abstract

Mesenchymal stromal cells (MSC) have been reported to improve bacterial clearance in preclinical models of Acute Respiratory Distress Syndrome (ARDS) and sepsis. The mechanism of this effect is not fully elucidated yet. The primary objective of this study was to investigate the hypothesis that the antimicrobial effect of MSC in vivo depends on their modulation of macrophage phagocytic activity which occurs through mitochondrial transfer. We established that selective depletion of alveolar macrophages (AM) with intranasal (IN) administration of liposomal clodronate resulted in complete abrogation of MSC antimicrobial effect in the in vivo model of *Escherichia coli* pneumonia. Furthermore, we showed that MSC administration was associated with enhanced AM phagocytosis in vivo. We showed that direct coculture of MSC with monocyte‐derived macrophages enhanced their phagocytic capacity. By fluorescent imaging and flow cytometry we demonstrated extensive mitochondrial transfer from MSC to macrophages which occurred at least partially through tunneling nanotubes (TNT)‐like structures. We also detected that lung macrophages readily acquire MSC mitochondria in vivo, and macrophages which are positive for MSC mitochondria display more pronounced phagocytic activity. Finally, partial inhibition of mitochondrial transfer through blockage of TNT formation by MSC resulted in failure to improve macrophage bioenergetics and complete abrogation of the MSC effect on macrophage phagocytosis in vitro and the antimicrobial effect of MSC in vivo. Collectively, this work for the first time demonstrates that mitochondrial transfer from MSC to innate immune cells leads to enhancement in phagocytic activity and reveals an important novel mechanism for the antimicrobial effect of MSC in ARDS. Stem Cells *2016;34:2210–2223*

**Significance Statement**

In the manuscript, we demonstrate that alveolar macrophages are critical for the bacterial clearance effect with MSC treatment in mouse *Escherichia coli* pneumonia. In addition, for the first time we report that human bone marrow derived mesenchymal stem cells (MSC) transfer their mitochondria to macrophages both in vivo and in vitro via tunneling nanotubes (TNT) and microvesicle secretion. This leads to enhanced macrophage phagocytosis and improved bioenergetics. Mitochondrial donation via direct cell contact presents a novel important mechanism of the antimicrobial effect of MSC in the conditions complicated by bacterial infections. Given these findings, we believe that this manuscript represents a significant advancement in the understanding of functional properties of MSC and provides additional evidence for their therapeutic potential in acute, inflammatory lung disease.

## Introduction

Acute respiratory distress syndrome remains the leading cause of disability and death in critically ill patients with a mortality rate of 25–40% depending on disease severity 1. Acute Respiratory Distress Syndrome (ARDS) has many clinical phenotypes with the most common causes being bacterial and viral pneumonia and sepsis. The main characteristic of ARDS pathophysiology is excessive pulmonary inflammation 2. Resident macrophages are key orchestrators of immune responses. Alveolar macrophages (AM) are the first line of innate immune cells in the distal respiratory tract responsible for detection and elimination of invading pathogens, as well as initiation of the early host immune response.

Mesenchymal stem (stromal) cells are self‐renewing multipotent adult stem cells which can be isolated from bone marrow and many other tissues and organs 3, 4. Under in vitro conditions, they can be differentiated into multiple cell types 5. These cells possess regenerative, immune‐modulatory, and antimicrobial properties. In recent years, MSC were proven to have protective effects in several preclinical models of ARDS including the *ex vivo* human lung perfusion model 6, 7, 8, 9, 10, 11, 12, 13, 14. These results have informed the design of clinical trials of MSC in ARDS. In two small randomized phase I studies, plastic adherent MSC were shown to be safe and well tolerated in patients with moderate to severe ARDS, and a phase II clinical trial powered for efficacy is currently ongoing 15, 16. Successful recovery of two patients with severe refractory ARDS has been reported after administration of MSC therapy on a compassionate basis 17.

Despite the rapid translation of preclinical models showing beneficial effects of MSC in the injured lung into clinical trials, the mechanisms by which these cells exert their anti‐inflammatory and restorative functions are still unclear. For rationale development of MSC based therapy, we need better understanding of their mechanisms of action within the injured lung.

Mechanisms by which MSC enhance microbial clearance are of particular importance, as acute inflammatory conditions such as ARDS are often complicated or caused by bacterial infections. Therefore, potential therapeutic agents, while reducing inflammation should not hinder the host's ability to combat infection. Remarkably, in the in vivo models of ARDS induced by live bacteria, MSC consistently demonstrate capacity not only to reduce inflammation but also to improve bacterial clearance. Our group has demonstrated that the antimicrobial effect of MSC in ARDS is partially mediated by their secretion of antimicrobial peptides and proteins 10 and also by the modulation of phagocytic capacity of host monocytes 9 and AM 11. This ability of MSC or MSC‐derived microvesicles to enhance phagocytic capacity of host innate immune cells has been reported by other investigators, but the mechanism of this effect has not been fully elucidated 18, 19, 20.

Although many studies have demonstrated that the beneficial effects of MSCs are dependent on paracrine mechanisms including microvesicle or exosome release 10, 12, 20, 21, 22, 23, there is an increasing body of evidence indicating that direct cell contact between MSC and lung epithelial or endothelial cells, allowing mitochondrial transfer, is also important. Islam et al. reported that mitochondrial transfer from MSC to alveolar type II epithelial cells (ATII) improved survival in a Lipopolysaccharide (LPS)‐induced pneumonia murine model through the restoration of the ATII bioenergetics profile. Direct cell contact through formation of Connexin 43 gap junctions between MSC and ATII was required for mitochondrial donation 24. Mitochondrial transfer from MSC to bronchial epithelial cells via tunnelling nanotubes (TNT) was protective in in vivo models of asthma and COPD 25, 26. Also, mitochondrial donation from MSC to endothelial cells through TNT was reported to be protective in an in vitro model of reperfusion injury 27. However, it is still unknown whether or not cell contact‐dependent mechanisms are involved in MSC mitochondrial transfer to innate immune cells and how mitochondrial transfer would influence their capacity to clear bacteria.

In this study, we tested the hypothesis that MSC can transfer their mitochondria to macrophages through TNT and that this would enhance macrophage phagocytic activity. Some of the results of these studies have been previously reported in the form of an abstract 28.

## Materials and Methods

See Supporting Information for detailed descriptions.

### Human Bone Marrow‐Derived Mesenchymal Stem Cells (MSC)

Human bone marrow‐derived MSCs were obtained from the Texas A&M Health Science Centre College of Medicine, Institute for Regenerative Medicine (Temple, Texas, US), a NIH repository. The cells met all the criteria for the classification as MSCs as defined by the International Society of Cellular Therapy 29. For inhibitor experiments using Cytochalasin B (Sigma‐Aldrich, Dorset, UK, www.sigmaaldrich.com), cells were incubated in complete α‐MEM supplemented with 1% Fetal Calf Serum (FCS) and 500 nM of inhibitor for 1.5–2 hours 5% CO_2_ and 37°C. Cells were then washed three times with Dulbecco's phosphate buffered saline (DPBS) prior to in vitro and in vivo studies.

### Human Monocyte‐Derived Macrophages (MDM) and MSC Direct Coculture

Before each experiment, MSC were trypsinized, counted, washed with sterile 1X DPBS, resuspended in RPMI‐1640 medium 1% FCS and added to the culture of primary human macrophages at a 1:20 MSC/MDM ratio. Cells were stimulated with LPS (*Escherichia coli* O111:B4, List Biological Laboratories (Campbell, California, www.listlabs.com) 10 ng/ml) or live *E. coli s*train K1 at MOI of 10 for 4 or 24 hours. Each experiment was performed in triplicate, using cells from at least three different donors of MDM from the Northern Ireland Blood Transfusion Service (NIBTS). Buffy coats donated by the NIBTS were used with ethical approval from the School Research Ethics Committee of Queen's University Belfast.

### Mitochondrial Isolation and Artificial Transfer

Mitochondrial isolation from MSC was performed using the mitochondrial isolation kit for cultured cells (Thermo Fisher Scientific (Paisley, UK, www.thermofisher.com)) according to manufacturer's instructions. Isolated mitochondria were resuspended in RPMI 1% FCS according to the final cell count of MSC, maintained on ice and used immediately for artificial transfer. Transfer of isolated mitochondria to MDM in vitro was performed according to Caicedo et al. 30.

### In Vivo *E. coli* Pneumonia Model

Mice were anaesthetized and instilled with 3.5 × 10^6^ CFU of *E. coli* K1 in the volume of 35 µl intranasally (IN). After 4 hours mice received MSC treatment (1 × 10^6^ cells/mouse) either intravenously (IV) through the tail vein in 100 µl of PBS, or intranasally in 35 µl of PBS (in case of intranasal administration, mice received gaseous (Isoflurane) anaesthesia for brief immobilization). Control mice were treated with the same volumes of PBS as a vehicle control. Mice were monitored and euthanized by an overdose of general anaesthesia 24 or 48 hours after infection and broncho‐alveolar lavage fluid (BALF) samples or lungs were collected for analysis. Route of administration did not affect MSC capacity to decrease severity of lung injury, decrease inflammation and improve bacterial clearance. Similar proportions of human MSC were recovered from BALF after IN and IV administration, indicating that MSC home to airspaces when given intravenously (Supporting Information Fig. S1A, S1C).

### Flow Cytometry

MSC were stained with 200 nM MitoTracker Deep Red (Thermo Fisher Scientific (Paisley, UK, www.thermofisher.com) for 45 minutes at 5% CO_2_ and 37°C before experiments. Mouse BALF cells or lung homogenate were stained with antibodies against CD11c (PE or APC), CD11b (APC‐e‐Fluor780), F4/80 (PE‐Cy7), Gr‐1 (e‐Fluor450 or PerCP‐Cy5, www.ebioscience.com) or appropriate IgG (all from eBiosciences, Hatfield, UK) and human macrophages were stained with anti‐CD45 (PE or APC) or appropriate IgG controls (eBiosciences). Cells were analyzed using a FACSCanto II flow cytometer and FlowJo software (Tree Star).

AM were gated as Gr‐1^−^F4/80^+^CD11c^hi^CD11b^low^, total lung macrophages were gated as Gr‐1^−^F4/80^+^, lung monocytes as Gr‐1^−^, CD‐11b^+^ and neutrophils as Gr‐1^+^ 31.

### Statistics

Data were tested for normality by using the D'Agostino and Pearson Omnibus normality test, Kolmogorov‐Smirnov test or the Shapiro‐Wilk test in GraphPad Prism 5. Comparisons of parametric data were analyzed by Student's *t*‐test, or one‐way or two‐way ANOVA for multiple groups, with post hoc analysis using the Bonferroni method. For nonparametric data the Mann‐Whitney *U* test was used for two group comparisons and Kruskal Wallis test for multiple groups with Dunn's post hoc correction. Statistical significance was considered when *p* < .05 and all data are displayed as mean ± SD. All statistical analysis was performed using GraphPad Prism version 5.

## Results

### Alveolar Macrophage Depletion Abrogates the Antimicrobial Effect of MSCs in an in vivo Model of *E. coli* Pneumonia

To model ARDS, we used a mouse acute *E. coli* pneumonia model, previously extensively characterized by our group 7, 10. To investigate the importance of AM as mediators of the effects of MSC in vivo, mice were selectively depleted of their alveolar macrophage population by intranasal administration of clodronate liposomes (CL) before infection (Supporting Information Fig. S2A, S2B). We compared the effects of MSC in normal and macrophage depleted mice after *E. coli* infection.

**Figure 1:**
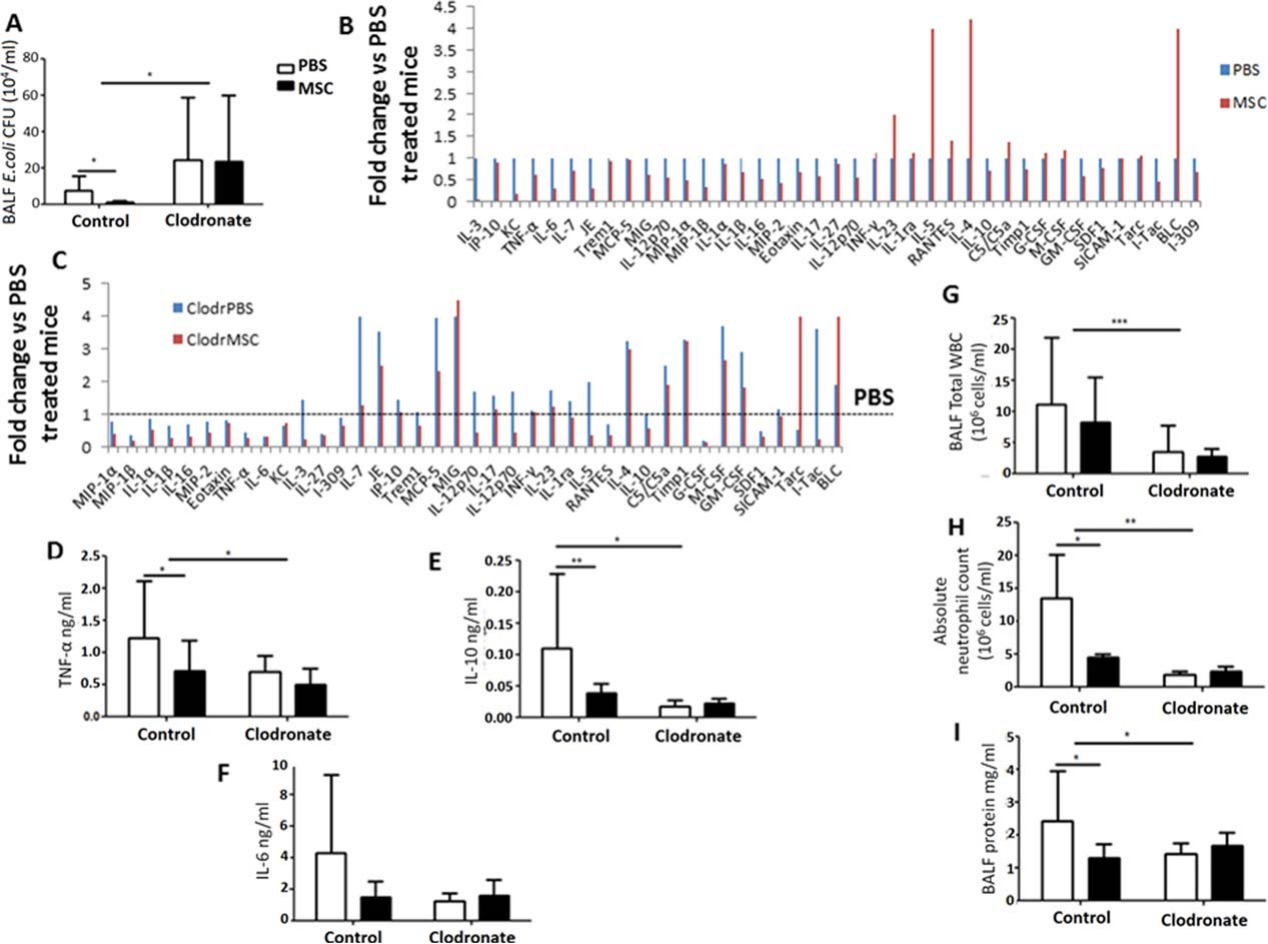
Effect of alveolar macrophage (AM) depletion on MSC antimicrobial and anti‐inflammatory properties in mouse *E. coli* pneumonia. **(A)** AM‐depleted mice had significantly higher *E. coli* CFU counts in BALF compared to nondepleted mice treated with PBS (*, *p* < .05 vs. control PBS, 2‐way ANOVA (Bonferroni)). MSC administration had no effect on bacterial clearance in the AM‐depleted group although significantly reducing *E. coli* CFU in control mice compared to PBS controls (*, *p* = .02, Student's *t*‐test). **(B)** Cytokine profile of BALF samples from normal mice. **(C)** Cytokine profile of BALF samples from AM‐depleted mice. **(D‐F)** AM‐depleted mice had significantly reduced levels of BALF TNF‐α, IL‐10, and IL‐6 compared to nondepleted animals treated with PBS. MSC administration had no effect on cytokine levels in AM‐depleted animals (*, *p* < .05 vs. control PBS, 2‐way ANOVA (Bonferroni)). MSC treatment significantly decreased TNF‐α and IL‐10 levels in nondepleted animals (*, *p* = .02 and **, *p* = .007 respectively vs. PBS treated group, Student's *t*‐test). **(G, H)** BALF total WBC counts and absolute neutrophil counts were significantly abrogated in the AM‐depleted group. MSC administration had no effect in AM‐depleted animals, while although reducing absolute neutrophil counts in nondepleted mice (***, *p* < .001, *, *p* < .05 vs. control PBS, 2‐way ANOVA (Bonferroni)). **(I)** BALF protein influx was significantly decreased in the AM‐depleted group versus nondepleted mice treated with PBS. MSC treatment significantly reduced BALF protein concentration in nondepleted mice and had no effect in AM‐depleted animals (*, *p* = .03, Student's *t*‐test). All data expressed as mean ± SD for each condition (at least *n* = 4 mice/condition). Abbreviations: BALF, broncho‐alveolar lavage fluid; MSC, mesenchymal stem cells; PBS, phosphate buffered saline; WBC, white blood cells.

Remarkably, AM‐depleted mice had twofold higher bacterial CFU numbers in the BALF compared to normal mice, and the antimicrobial effect of MSC administration was not present, whereas it was significant in nondepleted animals (Fig. 1A). This suggests that AM are key cellular mediators of the antimicrobial effect of MSC in this model.

The cytokine profile of the BALF was assessed by membrane‐based antibody array (R&D). In the case of normal mice, MSC demonstrated pronounced immunomodulatory effects (Fig. 1B). Levels of the majority of the pro‐inflammatory cytokines (MIP‐1α, MIP‐1β, IL‐1α, IL‐1β, IL‐16, MIP‐2, Eotaxin, TNF‐α, IL‐6, KC, IL‐3, IL‐27, I‐309, IL‐7, JE, IP‐10, Trem1, MCP‐5, MIG, IL‐12p70, IL‐17) were downregulated by MSC compared to the PBS treated group, whereas levels of several anti‐inflammatory mediators (IL‐4, IL‐5, RANTES) were elevated. Notably, IL‐10 levels were reduced by MSC treatment in normal mice as compared to PBS treated group. AM depletion resulted in reduction of levels of major pro‐inflammatory cytokines (MIP‐1α, MIP‐1β, IL‐1α, IL‐1β, IL‐16, MIP‐2, Eotaxin, TNF‐α, IL‐6, KC, IL‐27, I‐309) as compared to the nondepleted PBS treated group, suggesting that AM are important sources of these cytokines in this model. MSC treatment of AM‐depleted mice was not effective in restoring the levels of cytokines to those observed in the normal mice, suggesting that AM are important mediators of the immunomodulatory effect of MSC (Fig. 1C**)**. Results of the antibody array were validated by ELISA for TNF‐α, IL‐10 and IL‐6 (Fig. 1D–1F). In agreement with pro‐inflammatory cytokine data, inflammatory cell infiltration (assessed by total white blood cell counts and absolute neutrophil counts) and protein influx into the alveolar spaces were dramatically reduced after AM depletion, and MSC treatment had no effect on those parameters, whereas it significantly reduced both in normal mice (Fig. 1G–1I). Collectively, these data highlight a key role of AM in orchestrating the innate immune response in the alveoli and their importance as cellular mediators of MSC therapeutic effects in this in vivo model.

### Neutrophil Depletion did not Impair MSCs Antimicrobial Effect in vivo

To test if the absence of the antimicrobial effect of MSC seen with AM depletion was due to impaired neutrophil recruitment, in separate experiments we depleted mice of neutrophils by repeated intraperitoneal injections of anti‐Ly6G Ab (1A8).

Neutrophil depletion resulted in a statistically significant increase in bacterial growth in the BALF as compared to nondepleted mice and this effect was partially abrogated by MSC administration, suggesting that neutrophils are not critical for the antimicrobial effect of MSC. (Supporting Information Fig. S3A). Overall, similar to AM depletion, neutrophil depletion demonstrated a trend toward reduction in severity of lung injury (as indicated by BALF protein) and MSC were capable of reducing it further (1.8 ± 1.8 mg/ml for MSC vs. 2.6  ± 1.9 mg/ml for PBS) (Supporting Information Fig. S3B). Interestingly, although not statistically significant, BALF levels of TNF‐alpha were almost 50% higher in neutrophil depleted mice as compared to control animals (2.6 ± 2.5 ng/ml PBS and 1.6 ± 1.6 ng/ml MSC for depleted group vs control group with 1.5 ± 1.4 ng/ml PBS and 0.75 ± 0.99 ng/ml MSC) (Supporting Information Fig. S3C), indirectly supporting the previous conclusion that macrophages are the main source of pro‐inflammatory cytokines in this model.

### MSC Increase Phagocytosis both in Mouse Alveolar Macrophages and Human Monocyte‐Derived Macrophages (MDM)

To further investigate the finding that macrophage depletion resulted in abrogation of the antimicrobial effect of MSC, we tested the effect of MSC on AM phagocytosis in vivo.

**Figure 2:**
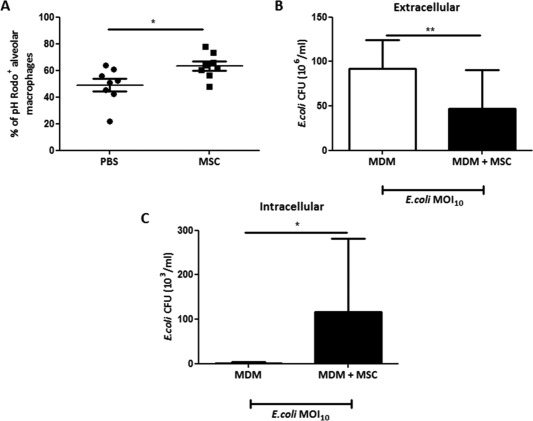
MSC enhance macrophage phagocytosis. **(A)** In the *in vivo E. coli* pneumonia model, MSC treatment significantly increased the percentage of alveolar macrophage positive for pHrodo *E. coli* bioparticles compared to PBS treated mice ((*n* = 7 mice/condition), *, *p* = .01, Student's *t*‐test). (B, C) *In vitro* human MDM were infected with *E. coli* (MOI 10) with or without direct coculture with MSC (1/20 ratio). **(B)** MSC coculture significantly reduced extracellular *E. coli* CFU counts coupled with **(C)** significantly elevated levels of intracellular CFU (*n* = 3 in triplicate, *, *p* < .05, **, *p*< .01, Student's *t*‐test). Data are shown as mean ± SD for each condition. Abbreviations: MDM, monocyte‐derived macrophage; MSC, mesenchymal stem cells; PBS, phosphate buffered saline.

To assess phagocytic activity of AM in vivo, BALF was harvested 24 hours after treatment with MSC or PBS, BALF cells were incubated with pHRodo‐conjugated *E. coli* particles and analyzed by flow cytometry. The AM population was identified as CD11c^hi^F4/80^+^CD11b^low^Gr‐1^−^ cells. BALF from the MSC treated group had a significantly larger population of phagocytic AM than BALF from PBS treated mice (Fig. 2A), suggesting MSC treatment enhanced alveolar macrophage capacity to engulf invading bacteria in the airway.

To extend this finding to a more clinically relevant human scenario and to elucidate the mechanisms of this effect, we cocultured MSC with primary human MDM in vitro. MSC and primary human MDM were cocultured together in direct contact for 4 hours. In the presence of MSC, macrophages increased extracellular *E. coli* bacterial killing by 80% (*p* = .03, *n* = 4) (Fig. 2B). The reverse effect was observed in the macrophage intracellular CFU counts, which were significantly increased with MSC, suggesting enhanced phagocytosis (Fig. 2C).

### MSC Transfer their Mitochondria to Macrophages in vitro and in vivo

**Figure 3:**
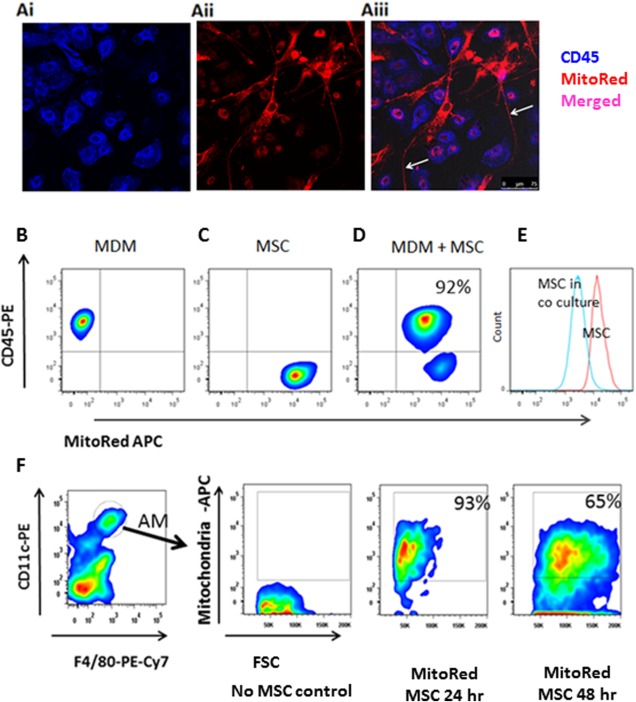
Mitochondrial transfer from MSC to macrophages. **(A)** Transfer of mitochondria from MSC to primary human macrophages through TNT‐like structures **(Ai)** human MDM uniformly express CD45 (blue) **(Aii)** MSC mitochondria are labeled with MitoRed (red) **(Aiii)** In coculture with MSC for 24 hours, colocalization of blue and red, indicates robust transfer of mitochondria from MSC to MDM. Network of mitochondria‐positive TNT emerging from the MSC and connecting to several distant macrophages (up to 200 µm) is also observed (arrows) (images were taken at a magnification of 10 × 63; scale bar = 50 µm). **(B)** Population of MDM cultured alone, stained with CD45‐PE but negative for MitoRed‐APC. **(C)** Population of MSC cultured alone, stained with MitoRed‐APC but negative for CD45‐PE. **(D)** After 4 hours in coculture, more than 90% of CD45^+^MDM demonstrate acquisition of MitoRed fluorescence (APC^+^), indicating extensive mitochondrial transfer from MSC. **(E)** Intensity of MitoRed fluorescence of MSC population decreased after coculture with MDM (blue histogram). Data representative of at least three independent experiments. **(F)** *E. coli*‐infected mice were treated with MitoRed‐labeled MSC IN, AM were gated as Gr‐1^−^F4/80^+^CD11c^hi^CD11b^low^ and analyzed for their expression of MitoRed fluorescence at 24 and 48 hours after treatment. Ninety‐three percent and sixty‐five percent of AM were positive for MitoRed at 24 and 48 hours, respectively. Plot representative of 3 mice/condition. Abbreviations: MDM, monocyte‐derived macrophage; MSC, mesenchymal stem cells.

A number of recent publications have shown that mitochondrial transfer from MSC to lung epithelial and endothelial cells is an important mechanism of MSC protective effects in several animal models of lung diseases 24, 25, 26, 27, 32. We hypothesized that mitochondrial transfer could be a mechanism by which MSC facilitate macrophage phagocytosis. MSC were labeled with 200 nM MitoTracker Deep Red for mitochondrial staining. Using immunofluorescent imaging, we observed that after 4 hours in coculture with MSC all MDM acquire MSC mitochondria (Fig. 3A). We were also able to visualize formation of intercellular cytoplasmic bridges termed tunnelling nanotubes (TNT) between MSC and macrophages staining positively for MSC mitochondria (Fig. 3A (arrows)), suggesting TNT as a mechanism of transfer. To rule out potential residual leakage of MitoTracker dye, MSC were washed three times before coculture and the excess media was tested instead of MSC (data not shown). We also did not observe any evidence of macrophage phagocytosis of MSC or MDM‐MSC cell fusion both by confocal and real‐time microscopy up to 24 hours in coculture, excluding the possibility that the acquisition of MSC mitochondria by macrophages was due to these mechanisms. These results were further corroborated by flow cytometry. Almost 100% of CD45‐positive MDM acquired MitoRed fluorescence specific for MSC mitochondria after 4 hours in coculture (Fig. 3B–3D). Notably, MitoRed Median Fluorescence Intensity (MFI) of MSC (CD45^neg^ MitoTracker^+^cells) had decreased approximately five‐fold in coculture as compared to MSC on their own, indicating the loss of fluorescence due to the transfer (Fig. 3E). Presence of a distinct CD45^neg^MitoRed^high^ population in coculture additionally confirmed that MSC were not phagocytosed by MDM.

To test if mitochondrial transfer from MSC to AM could be detected in vivo, we infected mice with *E. coli* as before and 4 hours later instilled MSC labeled with MitoRed IN. Twenty‐four and forty‐eight hours after infection lungs were harvested and lung homogenates analyzed by flow cytometry. Overall, at 24 hours, among the innate immune cells in the lung homogenate, the main recipients of MSC mitochondria were macrophages which we define as Gr‐1^–^F4/80^+^ cells (39 ± 9%) as compared to monocytes (Gr‐1^−^F4/80^−^CD11c^−^CD11b^hi^) (7 ± 3%) and neutrophils (Gr‐1^+^) (4 ± 1%). Remarkably, 96 ± 2% and 67 ± 7% of the alveolar macrophage population (Gr‐1^−^F4/80^+^CD11c^hi^ CD11b^low^) were positive for MitoRed fluorescence at 24 and 48 hours respectively (Fig. 3F) indicating effective and sustainable mitochondrial transfer in vivo.

### Mitochondrial Transfer Enhances Phagocytosis in Mouse Alveolar Macrophages in vivo and Primary Human Macrophages in vitro

**Figure 4:**
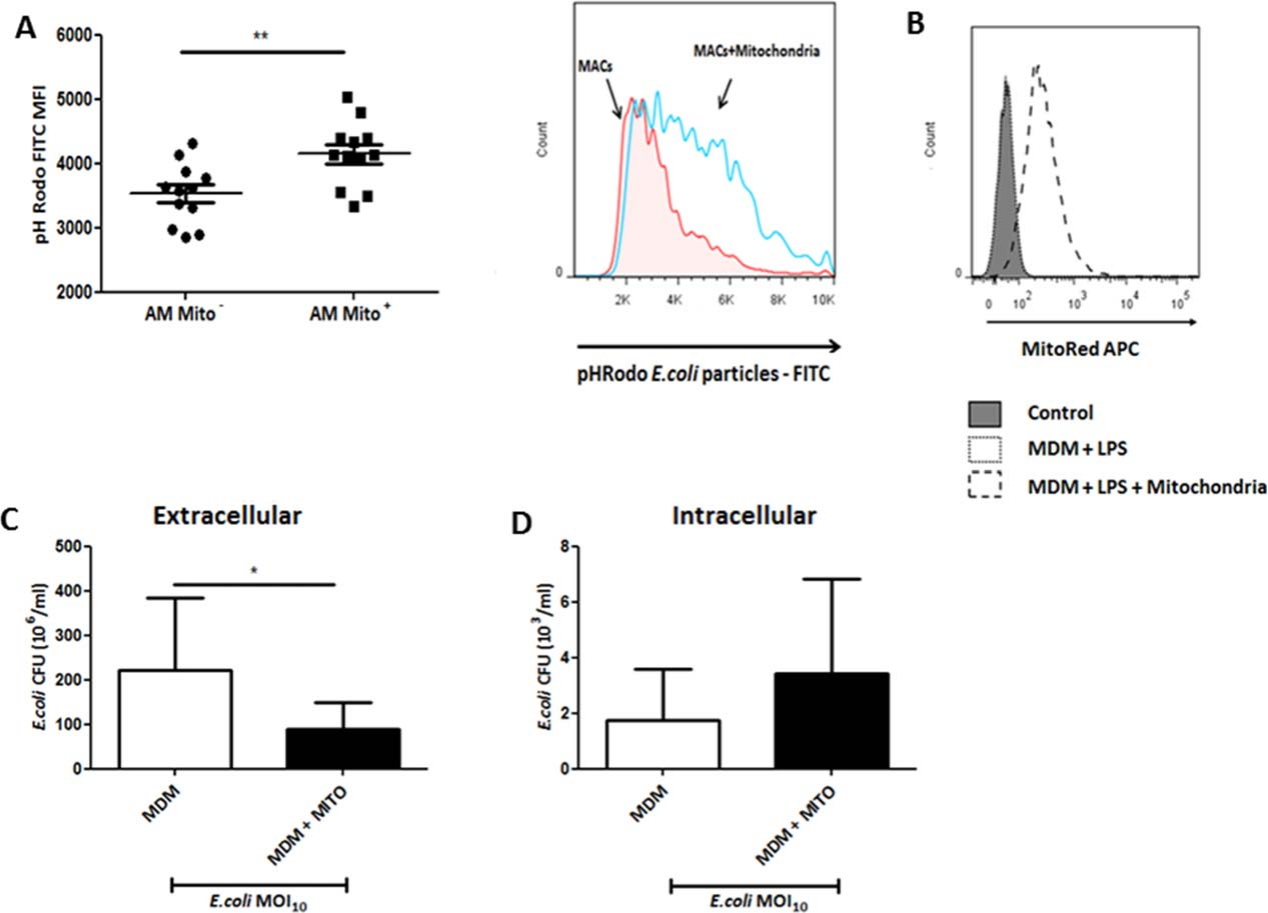
Internalized by macrophages, mesenchymal stem cells (MSC) mitochondria enhance their phagocytic activity. **(A)** In *E. coli* pneumonia MSC (MitoRed)‐treated mouse BALF was harvested and phagocytic activity of alveolar macrophage was assessed using fluorescent *E. coli* bioparticles by flow cytometry. Macrophages that had internalized MSC mitochondria (Mito+) showed a significantly higher phagocytic index in comparison to those without (Mito‐) (*n* = 12, **, *p* = .003, Student's *t*‐test). This was assessed by an increase in pHRodo median fluorescence intensity (MFI). **(B)** Isolated mitochondria taken from MitoRed‐treated MSC were added to human MDM and internalization was confirmed after 24 hours by flow cytometry. **(C)** *In vitro* addition of isolated MSC mitochondrial fraction to *E. coli* infected MDM significantly reduced extracellular CFU counts (*n* = 3 in triplicate, *, *p* < .05, Student's *t*‐test) coupled with an increase in intracellular CFU **(D)**. Abbreviations: MDM, monocyte‐derived macrophages; Mito, mitochondria.

To determine the effect of mitochondrial transfer on macrophage phagocytosis in vivo, mice were treated with MitoRed‐labeled MSC IN, BALF was harvested 24 hours after infection and phagocytic activity of BALF AM was assessed using pHRodo *E. coli* particles as before. Alveolar macrophages which had acquired MSC mitochondria had a significantly higher phagocytic index (measured by pHRodo MFI as compared to AM which did not have MSC mitochondria) (4,149 ± 507 for MitoRed^+^ AM vs. 3,528 ± 470 for MitoRed^−^ AM, mean MFI ± SD, *n* = 12, *p* = .003), suggesting that mitochondrial transfer is associated with enhanced phagocytic capacity (Fig. 4A).

**Figure 5:**
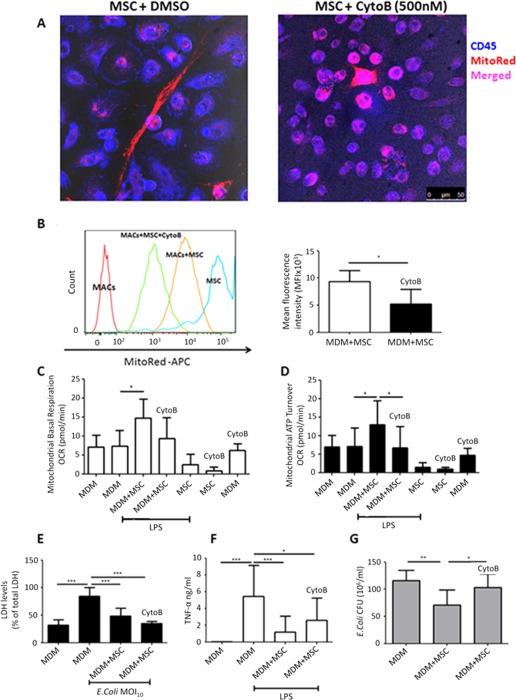
Inhibition of MSC tunneling nanotubes (TNT) formation by pretreatment with Cytochalasin B partially blocks mitochondrial transfer differentially affecting MSC modulation of MDM. **(A)** Confocal microscopy demonstrates normal spindle‐shape morphology of MitoRed MSC (red) in coculture with MDM (CD45+, blue), where TNT are present and mitochondrial transfer is evident (images were taken at a magnification of 10 × 63; scale bar = 50 µm). Cytochalasin B (500 nM) pretreated MSC appear rounded and TNT are no longer visible, however mitochondrial transfer still takes place as shown by colocalization of staining. **(B)** Coculture of MDM with Cytochalasin B pretreated MSC resulted in approximately 50% abrogation in the MitoRed MFI of macrophages (*, *p* < .05, Mann‐Whitney *U* test). **(C, D)** Mitochondrial respiration of human macrophages and human MSC was measured as oxygen consumption rate (OCR) using the SeaHorse technology. Macrophage mitochondrial function was analyzed during coculture with or without human MSC in the presence or absence of Oligomycin, FCCP, and Rotenone/Antimycin A to differentiate ATP‐linked respiration from proton leak. Coculture with untreated but not Cytochalasin B pretreated MSC significantly enhanced MDM levels of mitochondrial basal respiration and mitochondrial ATP turnover (*n* = 5–6, *, *p* < .05, Mann‐Whitney *U* test). **(E)** MSC pretreated with Cytochalasin B significantly restored cell viability of MDM post *E. coli* infection (***, *p* < .001 vs. MDM, ANOVA (Bonferroni)). **(F)** Both intact and Cytochalasin B pretreated MSC coculture significantly decreased LPS‐induced TNF‐α levels in culture medium (CM) (*, *p* < .05, **, *p* < .01, ***, *p* < .001 vs. MDM+LPS, **, *p* < .05 vs. MDM, ANOVA (Bonferroni). **(G)** Extracellular *E. coli* CFU were significantly decreased in coculture with untreated but not Cytochalasin B pretreated MSC compared to MDM alone (*, *p* < .05, **, *p* < .01 vs. MDM, ANOVA [Bonferroni]). Data shown as mean ± SD, *n* = 3–4 in triplicate for each condition. Abbreviations: MDM, monocyte‐derived macrophages; MFI, median fluorescence intensity; MSC, mesenchymal stem cells.

It has been reported previously that isolated MSC mitochondria are readily internalized by cancer cells, stay functional within the cell after artificial transfer and play a role in altering cancer cell bioenergetics 27. We decided to use isolated MSC mitochondria to confirm their role in macrophage phagocytosis in vitro. MSC mitochondria were isolated and added to human MDM culture for 24 hours before stimulation with live *E. coli* as in previous experiments. Internalization of isolated mitochondria by macrophages was confirmed by flow cytometry (Fig. 4B). Addition of isolated mitochondria to MDM resulted in a 60% decrease in the extracellular *E. coli* CFU as compared to MDM alone, mimicking the effect of MSC coculture (77% decrease in extracellular CFU) (Fig. 4C). Consistently, addition of isolated mitochondria led to an increase in intracellular *E. coli* CFU counts, similar to MSC coculture, compared to MDM alone suggesting improved phagocytosis (Fig. 4D). Islam et al. demonstrated that formation of gap junctions through Connexin‐43 was necessary for cell‐contact‐dependent mitochondrial transfer from MSC to alveolar epithelial cells 24. In the in vitro coculture we observed that mitochondria are transferred to MDM through TNT which form cell contacts with macrophages. Addition of GAP 26, a specific inhibitor for Connexin‐43 gap junction formation, did not alter either formation of TNT‐macrophage contacts or the rate of mitochondrial transfer (data not shown), ruling out involvement of Connexin‐43 based gap junctions. We later hypothesized that blocking of TNT formation in MSC would abrogate the transfer. Cytochalasin B at nanomolar concentrations has been reported to block the formation of TNT without affecting endocytosis and phagocytosis and has been used to block TNT formation in MSC previously 27, 33, 34. Preincubation of MSC with 500 nM Cytochalasin B resulted in inhibition of TNT formation and substantial changes in cell morphology (Fig. 5A), however mitochondrial transfer, although less intensive, was still evident (Fig. 5A, 5B). This would suggest that TNT mediate transfer only partially and macrophages also acquire MSC mitochondria through cell contact‐independent mechanisms.

Cytochalasin B pretreatment of MSC resulted in a near 50% reduction in MitoRed mean fluorescence intensity (MFI) in MDM as compared to MDM in coculture with untreated MSC (9,300 ± 2,068 vs. 5,240 ± 2,643 MDM MitoRed MFI, mean ± SD, *p* = .028) (Fig. 5B).

To further investigate if transferred mitochondria are functional, we examined macrophage mitochondrial respiration and mitochondrial ATP turnover using SeaHorse technology 35. Remarkably, coculture with MSC led to a significant and robust increase in both MDM mitochondrial basal respiration rate and mitochondrial ATP turnover as measured by oxygen consumption rate (OCR). Coculture with Cytochalasin B pretreated MSC abrogated this effect (Fig. 5C, 5D), confirming that TNT mediate transfer of functional mitochondria and that this process can be blocked by Cytochalasin B pretreatment.

In coculture with MDM, Cytochalasin B pretreated MSC were capable of rescuing MDM from *E. coli*‐induced cell death to a similar extent as nontreated MSC (50 and 40% respectively) (Fig. 5E) and retained the significant capacity to suppress LPS‐induced TNF‐α secretion by MDM, although less potently than untreated MSC (Fig. 5F). This provides additional proof that the secretory function of MSC was not significantly affected by Cytochalasin B pretreatment. However, Cytochalasin B pretreated MSC completely lost the capacity to enhance bacterial clearance as seen with untreated MSC (Fig. 5G), indicating that partial abrogation of mitochondrial transfer is responsible for the MSC effect on macrophage phagocytosis in vitro.

Thus, mitochondrial transfer through TNT resulted in improvement of MDM phagocytosis potentially through enhancement of MDM mitochondrial function and ATP turnover. Importantly, by Cytochalasin B pretreatment we were able to selectively block the effect of MSC on macrophage phagocytosis and bioenergetics without compromising their anti‐inflammatory properties.

### Partial Inhibition of Mitochondrial Transfer by Prevention of TNT Formation in MSC Abrogates the Antimicrobial Effect of MSC in *E. coli* Pneumonia

**Figure 6:**
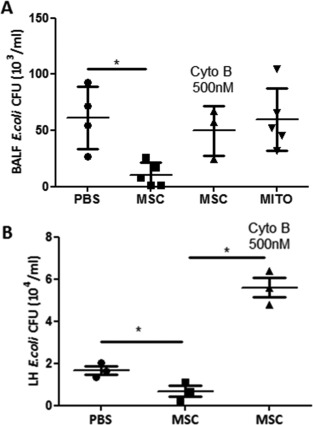
Inhibition of tunneling nanotubes formation by MSC abrogates the antimicrobial effect of MSC in *E. coli* pneumonia. Pretreatment of MSC with Cytochalasin B inhibited the therapeutic effect of MSC in bacterial clearance in the BALF **(A)** and lung homogenate **(B)**. Administration of the MSC mitochondrial fraction did not significantly affect the bacterial burden in the BALF compared to the PBS group (in BALF *, *p* < .05 MSC vs. PBS, Kruskal Wallis test, in lung homogenate *, *p* < .05 MSC vs. CytoB, Kruskal Wallis test, PBS vs. MSC, Student's *t*‐test, *n* = 3–5 mice per group). Data shown as mean ± SD for each condition. Abbreviations: BALF, broncho‐alveolar lavage fluid; MSC, mesenchymal stem cells.

To extend this finding to an in vivo setting, we treated mice with untreated, Cytochalasin B pretreated MSC and MSC isolated mitochondria. Pretreatment of MSC with Cytochalasin B completely abrogated the antimicrobial effect of MSC both in the BALF and lung homogenate resulting in a nearly twofold increase of *E. coli* CFU in the BALF, similar to the effect seen with AM depletion (Figs. 1A, 6A, 6B). This would indicate the importance of cell‐contact‐dependent mitochondrial transfer for the antimicrobial effect of MSC in vivo. The antimicrobial effect of MSC was not recapitulated by the administration of MSC isolated mitochondria, suggesting that intact MSC are required for efficient transfer of mitochondria which may functionally integrate into the recipient cell in vivo.

### Cell Contact‐Independent Mitochondrial Transfer

**Figure 7:**
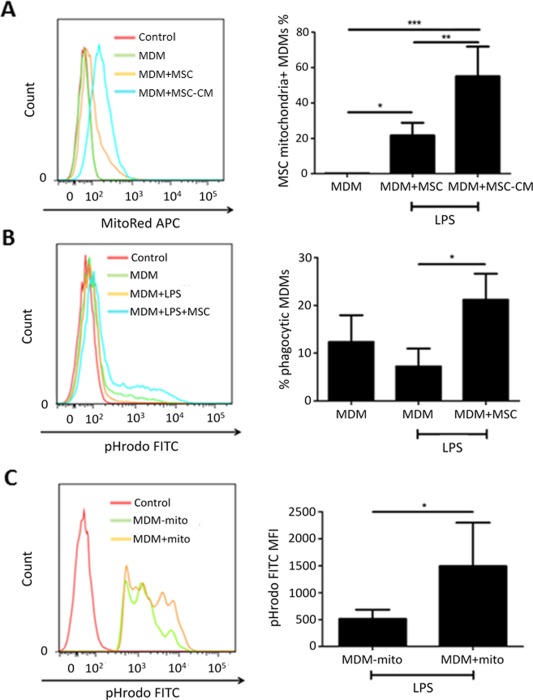
Mitochondrial transfer from MSC to human MDM via noncontact dependent mechanism. MDM were cocultured with MSC‐CM or MSC (pretreated with MitoRed) in a Transwell system without cell contact for 24 hours in the presence of LPS. **(A)** The extent of mitochondrial transfer to MDM was measured by flow cytometry (*n* = 3–5/group, *, *p* < .05, **, *p* < .01, ***, *p* < .001, ANOVA [Bonferroni]) **(B)** The MDM were also given pHRodo particles to quantify phagocytosis (*n* = 5/group, *, *p* < .05, ANOVA (Bonferroni)). **(C)** Phagocytic MDM were divided into two groups, with and without internalization of MSC mitochondria, and their phagocytic indexes were determined by median fluorescence intensity (MFI) (*n* = 5/group *, *p* < .05, Student's *t*‐test). Abbreviations: CM, culture medium; MDM, monocyte‐derived macrophages; MSC, mesenchymal stem cells.

Although TNT‐mediated mitochondrial transfer was essential for the MSC effect on phagocytosis both in vitro and in vivo, we also explored the importance of exosome‐mediated mitochondrial transfer. MSC were prelabeled with MitoTracker Red and cocultured with human MDM at a 1/5 ratio in the Transwell noncontact coculture system. The higher MSC number was implemented to compensate for the distance which the exosomes would have to travel through the inserts to reach the MDM. After 24 hours, 21.7 ± 5.9% of macrophages had acquired MSC mitochondria with coculture and this was increased to 55.1 ± 16.8% with the use of MSC conditioned medium (Fig. 7A). This would suggest effective transfer albeit less potent than the extent of transfer when cells were cocultured in direct contact. Consistent with previous findings, MSC in noncontact coculture significantly enhanced the proportion of phagocytic macrophages when stimulated with LPS as quantified using pHRodo particles (Fig. 7B), and phagocytic macrophages which had internalized MSC mitochondria demonstrated a higher phagocytic index than those without mitochondria (Fig. 7C).

## Discussion

The main findings in this study are that (1) the antimicrobial effect of MSC in a mouse model of *E. coli*‐induced pneumonia is dependent on alveolar macrophages; (2) MSC transfer their mitochondria to macrophages both in vitro and in vivo; (3) mitochondrial transfer from MSC to primary human MDM is at least partially mediated through TNT‐like structures; (4) mitochondrial transfer from MSC to macrophages improves macrophage mitochondrial function and ATP turnover in vitro and enhances macrophage phagocytic capacity both in vitro and in vivo and (5) TNT‐mediated mitochondrial transfer from MSC to alveolar macrophages is an important mechanism of the antimicrobial effect of MSC in vivo.

AM are considered to play a prominent role in clearing bacterial infections from the airspaces by phagocytosis of airborne microorganisms and by orchestrating inflammatory responses. Although beneficial effects of MSC treatment in animal models of *E. coli* pneumonia are well documented 7, 10, 20, 36 the role of AM as cellular mediators of the MSC effect has not been directly addressed. Therefore, in this study mice were depleted of AM by the IN instillation of clodronate‐containing liposomes before infection with *E. coli* K1. AM depletion resulted in downregulation of levels of major pro‐inflammatory cytokines as well as decreased recruitment of inflammatory cells (neutrophils and monocytes) into the airspaces, associated with pronounced impairment in bacterial clearance at 24 hours post infection. Notably, the antimicrobial effect of MSC was completely abrogated with AM depletion (Fig. 1A). To test if the loss of the MSC antimicrobial effect could be a consequence of compromised neutrophil recruitment, we depleted mice of neutrophils and found that although neutrophil depletion resulted in a significant increase in BALF bacterial burden, MSC treatment partially abrogated this effect (Supporting Information Fig. 3A). Collectively, these findings suggest that AM but not neutrophils are essential for the antimicrobial effect of MSC in *E. coli* pneumonia model.

One of the potential mechanisms by which MSC could enhance bacterial clearance is to enhance host phagocytes capacity to engulf and kill invading microorganisms. We have reported previously that MSC improved phagocytic capacities of peripheral blood monocytes in the model of *P. aeruginosa peritonitis* 9 and also of AM in the model of *E. coli*‐induced pneumonia in the *ex vivo* perfused human lung preparation 11. These findings were further confirmed by Devaney et al. 36, showing that the MSC beneficial effect in rat *E. coli* pneumonia was associated with increased phagocytic activity of monocytes and macrophages and also by Monsel et al. 20, who demonstrated that MSC or MSC‐derived microvesicles improved phagocytic capacity of primary human monocytes. In agreement with these findings, our present work demonstrates that MSC administration was associated with a significantly increased number of phagocytic AM compared to control mice (Fig. 2A). These data were further corroborated in vitro, where coculture with MSC led to significant improvement in the capacity of primary human MDM to eliminate extracellular bacteria (Fig. 2B). Interestingly, Hall et al. 18 reported that the antimicrobial effect of mouse MSC in the cecal ligation and puncture sepsis model was mediated through improvement of neutrophil phagocytosis, and neutrophil depletion was detrimental to the beneficial effects of MSC. However, in our studies we were not able to detect any differences in neutrophil phagocytosis with MSC 9, these inconsistencies might be due to different models used by our groups as well as differences in the functional properties of human and mouse MSC.

It is generally considered that the secretion of paracrine factors is one of the primary mechanisms of MSC effect 37. Although in many studies it has been reported that MSC cell products (conditioned medium or exosomes) were able to recapitulate effect of the cells 20, 22, 23, 38, 39, 40, there are also reports showing that the MSC secretome was not effective 36 or was not as effective as whole cell therapy 41, suggesting a role for cell‐contact‐dependent mechanisms. Direct cell‐contact‐dependent mitochondrial transfer from MSC to lung epithelial and endothelial cells has been reported as an important mechanism of MSC beneficial effects in several preclinical animal models of lung diseases 20, 24, 25, 26. The ability of MSC to transfer their mitochondria to innate immune cells via cell‐contact‐dependent mechanisms has not been studied yet. In our experiments, we found that MSC possess a profound capacity to transfer their mitochondria to macrophages both in vitro and in vivo (Fig. 3). Furthermore, we visualized that transfer occurs through TNT‐like structures which are formed by MSC. TNT were first described by Rustom et al. in 2004 42 as a novel mechanism of cell‐cell communication. Since then numerous reports have demonstrated that TNT facilitate the exchange of signaling molecules and organelles, including mitochondria between connected cells 43, 44, 45, 46. The TNT‐mediated mechanism of mitochondrial transfer from MSC to bronchial epithelial cells was shown to account for MSC beneficial effects in preclinical models of asthma and COPD 26. Furthermore, Miro1 motor protein has been shown to play critical role in mediating the transfer along the actin fibres within TNT 25. Islam et al., 2012 has reported that for MSC mitochondrial donation to alveolar epithelial cells, Connexin‐43 based gap junctions between MSC and recipient cells were required 24. In our work however, blocking Connexin‐43 gap junction formation by GAP 26 had no effect on the number of cell contacts between MSC‐derived TNT and macrophages or mitochondrial transfer rate.

Consistent with existing literature 23, 24, 26, we found that TNT‐mediated mitochondrial transfer augmented macrophage bioenergetics (basal respiration and mitochondria ATP turnover) confirming that transferred mitochondria were functional (Fig. 5C, 5D).

More significantly, we observed that AM that had acquired MSC mitochondria display higher phagocytic activity in vivo (Fig. 4A). To further test our hypothesis that mitochondria donation will enhance phagocytic activity of macrophages, we isolated the mitochondrial fraction from MSC and added it to macrophages in culture. Several reports have shown that isolated mitochondria can be directly internalized by cells, incorporated into the endogenous mitochondrial network and contribute to changes in the bioenergetic profile as well as functional properties of recipient cells, not only in vitro but also in vivo 48, 49. In our work, addition of the isolated MSC mitochondrial fraction to macrophages in vitro significantly improved their phagocytic activity similar to the effect of MSC coculture (Fig. 4C, 4D). By flow cytometry we observed internalization of exogenous mitochondria into the macrophage (Fig. 4B).

The mechanism of internalization of the artificially isolated mitochondria is as yet unknown. One could speculate that it would differ from the process occurring when mitochondria are being transferred naturally through TNT or exosomes and that may result in different functional outcomes for the recipient cell. This represents one limitation for this method as a gain of function approach to test the importance of MSC mitochondria in this effect.

Interestingly, when MSC formation of TNT was inhibited by Cytochalasin B, mitochondrial transfer was not abrogated completely, suggesting involvement of cell‐contact independent mechanisms most likely through microvesicle release by MSC (Fig. 5A, 5B). We have shown that even when MSC and MDM are cocultured without contact, MDM do acquire MSC mitochondria although to a substantially lesser extent than is observed with direct cell contact. Again this transfer is associated with an increased phagocytic capacity (Fig. 7). The capacity of MSC to release mitochondria–containing microvesicles has already been reported by Islam et al. (24) and further corroborated by Phinney et al. (23), who demonstrated that MSC outsource their partially depolarized mitochondria to macrophages through secretion of extracellular vesicles which are being engulfed by acceptor macrophages. This contributes to improvement of macrophage bioenergetics whereas another type of MSC shed exosomes simultaneously inhibiting macrophage activation by suppression of Toll‐like receptor signaling. This phenomenon may explain the capacity of MSC microvesicles to improve monocyte phagocytosis, shown by Monsel et al. 20 and also provides an additional explanation for the independence of the immunomodulatory effect of MSC on macrophages from mitochondrial transfer donation that we see in our study.

Finally, partial blockage of mitochondrial transfer by preincubation of MSC with 500 nM Cytochalasin B resulted in complete abrogation of the MSC effect on macrophage bioenergetics and clearance of extracellular bacteria while not affecting MSC pro‐survival and anti‐inflammatory properties in vitro (Fig. 5). Importantly, mice which were treated with Cytochalasin B pretreated MSC in vivo demonstrated significantly higher (fivefold to tenfold) bacterial burden in the lungs shown by BALF and lung homogenate *E. coli* CFU counts than mice which received normal MSC (Fig. 6A, 6B), similar to the effect seen with AM depletion (Fig. 1A). Of note, although we observed that addition of the mitochondrial fraction isolated from MSC to the macrophages in culture led to internalization and improved phagocytosis in vitro, administration of isolated mitochondria to mice in vivo did not have any effect on bacterial clearance (Fig. 6A). One possible explanation for that would be that the dose required for this effect in vivo would be different from the in vitro scenario. Also, we did not test the distribution of these exogenous mitochondria in the lung, it is possible that by IN route of administration mitochondria are not distributed evenly but are concentrated in one small area, which prevents their uptake by AM from distant alveoli and the IV route of administration could be more beneficial.

A recently published paper by Braza et al. 50 reports that in a house dust mite‐induced asthma model MSC are being phagocytosed by AM leading to an M2 phenotypic switch and alleviation of inflammation. In the present study we have tested the possibility of MSC phagocytosis by human MDMs in vitro by real‐time imaging and confocal microscopy and did not detect any evidence of phagocytosis or cell fusion up to 72 hours in coculture. It is possible that some apoptotic MSC will be phagocytosed by AM in vivo thereby accounting for some mitochondrial transfer, however the loss of MSC antimicrobial effect after inhibition of TNT‐like structures indicates the importance of active mitochondrial transport rather than passive phagocytosis for MSC modulation of AM in this model.

There are limitations to this study. This study is focused on the importance of MSC mitochondrial transfer to macrophages as a novel mechanism of the antimicrobial effect of MSC in vivo. We do not report here the effect of the transfer on other macrophage functions (e.g., cytokine and chemokine secretion or polarization); this is the main subject of on‐going work. The question still remains about the mechanisms by which MSC mitochondria facilitate macrophage phagocytosis. Mitochondria are implicated in the synthesis of high‐energy phosphates, modulation of calcium stores, activation of signaling pathways that impact cell fate as well as shuttling genetic material. It is plausible that mitochondrial transfer replenishes the ATP pool which is being quickly depleted by macrophages during cytoskeletal rearrangements in the process of phagocytosis. Although we have shown that mitochondrial transfer augments macrophage bioenergetics, we have not demonstrated that this is a direct cause for more active phagocytosis; this will require more in depth investigation utilizing MSC with dysfunctional mitochondria.

## Conclusion

In conclusion, MSC transfer their mitochondria to macrophages both in vitro and in vivo. Mitochondrial donation results in enhancement of macrophage phagocytosis potentially through improvement in bioenergetics and presents a novel mechanism of the antimicrobial effect of MSC in conditions complicated by bacterial infections.

## Author Contributions

M.J.: Conception and design, collection and assembly of data, data analysis and interpretation, manuscript writing. T.M.: Collection and assembly of data, data analysis and interpretation, manuscript writing. D.D.: Collection of data, technical assistance with animal studies. D.M.: Provision of the study material, manuscript writing. M.M.: Data analysis and interpretation, manuscript writing, financial support. A.K.: Data analysis and interpretation, manuscript writing. C.O.: Data analysis and interpretation, manuscript writing, provision of the study material. A.D.K. Conception and design, financial support, collection and assembly of data, data analysis and interpretation, manuscript writing, final approval of the manuscript.

## Disclosure of Potential Conflicts of Interest

The authors indicate they have no potential conflicts of interest.

## Supporting information

Supporting Information 1

Supporting Information Figure S1

Supporting Information Figure S2

Supporting Information Figure S3

Supporting Information Figure S6

Supporting Information
