# Supplementary material for: Mitochondrial Transfer via Tunneling Nanotubes is an Important Mechanism by Which Mesenchymal Stem Cells Enhance Macrophage Phagocytosis in the In Vitro and In Vivo Models of ARDS
Source: Stem Cells. 2016 Apr 29;34(8):2210–23. doi: 10.1002/stem.2372 (PMC4982045; doi:10.1002/stem.2372)
Supplement: Supplementary file 1 — Supporting Information 1 [file STEM-34-2210-s001.docx]

**Supplemental Materials and Methods**

*Animals*

For neutrophil depletion experiments C57BL/6 male mice (8- to 10-weeks old; Jackson Laboratory, USA) were used. Animals were maintained in the animal facility at the University of California, San Francisco (UCSF). All experimental protocols were approved by the Institutional Animal Care and Use Committee at UCSF.

For all other experiments C57BL/6 male mice (8- to 10-weeks old; Harland Institute, UK) were used. Animals were maintained in the Biological Services Unit (BSU) at the Queen’s University Belfast. Experiments were sanctioned and approved by the UK Home Office and Queen’s University Belfast Ethical Review Committee.

*Cell Culture*

*Isolation of human mononuclear cells and macrophage maturation*

Single donor leukocyte buffy coats were obtained from the Blood Transfusion Centre in Belfast (NIBTS) or from volunteers following ethical approval. Blood was diluted 1:2 with sterile HBSS (Hank’s buffered salt solution) (Thermo Fisher Scientific (Paisley, UK)) and mixed by inversion. The diluted blood sample was then layered gently onto 15 ml of Ficoll-Paque (Sigma Aldrich, Dorset, UK) at a 45^o^. They were then centrifuged at 480 g, 20^o^C for 20 mins without brake. The lighter mononuclear cells (monocytes and lymphocytes) sedimented to the plasma-density gradient interface and produced a pearlescent layer (buffy layer). This layer was then extracted, washed in HBSS and pelleted by centrifugation at 153 g, 4^o^C for 5 mins (with brake) at least three times. Mononuclear cells were then re-suspended in RPMI 1640 (Thermo Fisher Scientific (Paisley, UK)) supplemented with 1% heat-inactivated FCS (RPMI 1% FCS) (Thermo Fisher Scientific (Paisley, UK)). The cells were counted, seeded at 3x10^5^cells/ml and incubated at 37°C, 5% CO_2_ and 95% air in a humidified incubator for 1-2 hours. After this time, non-adherent cells were removed by washing twice with HBSS and media was replaced with RPMI supplemented with 10% heat treated FCS (RPMI 10% FCS), 10ng/ml GM-CSF (Peprotech, NJ, US). Cells were left to rest 5-7 days before subsequent experiments.

*Human bone marrow-derived mesenchymal stem cell (MSC) culture*

Allogeneic BM-derived human MSC were cultured as previously described (20). Briefly, MSC were obtained from the Texas A&M Health Science Centre College of Medicine, Institute for Regenerative Medicine (Temple, Texas), a NIH repository. The cells met all the criteria for the classification of MSC as defined by the International Society of Cellular Therapy (26). Cells were thawed and expanded in tissue culture flasks (BD Falcon, Belgium) at a density of 500,000 cells/175 cm^2^. Once cells had reached 70%–80% confluency they were passaged every 3-4 days by trypsinisation and were used for experiments between passages 2-5. MSC were cultured in α-Minimum Essential Medium (α-MEM) without ribonucleosides or deoxyribonucleosides containing 2mM L-glutamine and 16.5% FBS, 1% Penicillin/Streptomycin (complete α-MEM). Cells were cultured in a humidified incubator at 5% CO_2_ and 37°C under sterile conditions.

For inhibitor experiments using Cytochalasin B (Sigma-Aldrich, Dorset UK), cells were incubated in complete α-MEM supplemented with 1% FCS and 500nM of inhibitor for 1.5-2 hours 5% CO_2_ and 37°C. Cells were then washed three times with DPBS (Thermo Fisher Scientific (Paisley, UK)) prior to *in vitro* and *in vivo* studies.

*MDM and MSC co-culture*

Before each experiment, MSC were trypsinised, counted, washed with sterile 1x Dulbecco’s phosphate buffered saline (DPBS), re-suspended in RPMI-1640 medium 1% FCS and directly added to the culture of primary human macrophages at a 1:20 MSC/MDM ratio. For non-contact co-culture, cells were cultured in a Transwell (Corning, Flintshire, UK) system at the ratio of 1/5 MSC/MDM. Cells were stimulated with LPS (*E.coli* O111:B4, List Biological Laboratories Campbell, California) (10 ng/ml) or live *E.coli s*train K1 at MOI of 10 for 4 or 24 hr. Each experiment was performed in triplicate, using cells from at least three different donors of MDM from the NIBTS.

*Bacterial Culture and Phagocytosis assays*

*E.coli* strain K1 was used for subsequent experiments. The methods used to passage, store, amplify, and quantify the bacteria were performed as described previously (43). For each experiment, *E. coli* colonies were seeded from frozen stocks, and grown overnight at 37°C in liquid Lennox LB medium (Sigma Aldrich, Dorset, UK) with slight agitation. Before each experiment, the bacterial cells were washed once with sterile 1x PBS and re-suspended using RPMI 1% FCS. Optical density (OD at λ= 600 nm) of the suspension was measured. Number of CFU was calculated as according to the following equation: OD600= 0.9-1.0 corresponds to ~2 × 10^8^ CFU/ml. MDM were infected with *E.coli* (MOI 10) with or without MSC in direct contact. Plates were centrifuged at 350g for 5 mins and incubated in humidified incubator 5% CO_2_ and 37°C under sterile conditions for 4 or 24 hours. After this period, supernatants were collected for ELISA cytokine analysis, and total CFU was taken per condition for extracellular bacterial counts. MDM were washed with 1x PBS three times and incubated with 100µl RPMI 1% FCS supplemented with 300µg/ml of Gentamycin at 37^0^C for 45 mins. Cells were then lysed with 100µl 0.5% Saponin for 5 mins. 900µl 1x PBS was added and 50µl was plated for total intracellular CFU. 0.5% Saponin was found not to have bactericidal effects on *E.coli.*

*Mitochondrial isolation and artificial transfer*

Mitochondrial isolation from MSC was performed using the mitochondrial isolation kit for cultured cells from Thermo Fisher Scientific (Paisley, UK) according to manufacturer’s instructions. Isolated mitochondria were re-suspended in RPMI 1% FCS, maintained on ice and used immediately for the artificial transfer. Transfer of isolated mitochondria to MDM *in vitro* was performed according to Caicedo *et al*, 2015. Before isolation MSC were counted and used at a 1:20 ratio to MDM for intact cells, however mitochondria were added at a 1:40 ratio.

*In vivo E. coli pneumonia model*

Mice were anaesthetised and instilled with 3.5 x10^6^ CFU of *E. coli* K1 in the volume of 35µl intranasally (IN). After 4 hours mice received MSC treatment (1 x 10^6^ cells/mouse) either intravenously (IV) through the tail vein in 100µl of PBS, or IN in 35µlof PBS (in case of intranasal administration mice received gaseous (Isoflurane) anaesthesia for brief immobilisation). Control mice were treated with the same volumes of PBS as a vehicle control. Mice were monitored and euthanized by an overdose of general anaesthesia 24 hr or 48 hr after infection. For broncho-alveolar lavage (BAL), a small incision on the trachea was made, a 20-gauge needle was placed into the opening and secured in position using Mersilk suture (Ethicon, Scotland, UK). 1ml of sterile 1x PBS was flushed in and withdrawn 5 times in small increments. Broncho-alveolar lavage fluid (BALF) was collected, aliquots taken for bacterial CFU analysis, the rest was centrifuged at 425g for 10 min at 4°C and supernatants stored at -80°C for protein and cytokine measurements. Cell pellets were resuspended in 100μl of ice-cold PBS and used for cytospins, white blood cell counts, flow cytometry and phagocytosis assays. Total viable cell counts and total white blood cell counts were determined using a Countess automatic cell counter using trypan blue exclusion. In separate experiments, lungs were harvested and lung homogenate was prepared by homogenisation at the lowest setting, and used for bacterial CFU counts, flow cytometry and phagocytosis assays.

*Depletion of alveolar macrophages (AM) using clodronate liposomes*

Clodronate liposomes were purchased from www.clodronateliposomes.org. In brief, 50μl of liposomes was instilled IN 48 hr before *E.coli* infection and MSC treatment. Depletion of BALF CD11^hi^ F4/80^+^ AM was assessed by flow cytometry as described previously to determine percentage of depletion (~90% depletion**, Supplemental Figure 2**). As liposomes themselves might compromise phagocytic function of macrophages we did not use PBS containing liposomes in our control animals, but used PBS instead (28).

*Depletion of neutrophils using anti-Ly6G 1A8 Ab*

Neutrophils were depleted by 3 consecutive intraperitoneal (IP) injections of monoclonal anti-Ly6G 1A8 Ab (UCSF, Monoclonal Antibody Core), 500μg/mouse every 24 h and the percentage of depletion was assessed by measurement of neutrophil concentrations in the blood using Hemavet (Drew Scientific, FL, US). Only mice which had >80% neutrophil depletion on the day of experiment were entered into the study.

*Flow cytometry*

MSC were stained with 200nM of MitoTracker Deep Red (Thermo Fisher Scientific, Paisley, UK) for 45 mins at 5% CO_2_ and 37°C before experiments. Mouse BALF cells or lung homogenate were stained with antibodies against CD11c (PE or APC), CD11b (APC-e-Fluor780), F4/80-PECy7, Gr-1-(e-Fluor450 or PerCP-Cy5) or appropriate IgG (all from eBiosciences, Hatfield, UK). Human macrophages were stained with anti-CD45 (PE or APC) or appropriate IgG controls (eBiosciences). For comparison of IN and IV routes, pre-stained (MitoTracker) (**Supplemental Figure 1**) MSC were additionally stained with anti-human CD90 or appropriate IgG controls (eBiosciences, Hatfield, UK). In brief, after Fc-receptor blockage with anti-mouse CD16/32 or human Fc-receptor blockage (eBiosciences) cells were stained with the Ab cocktail as per the manufacturer’s instructions for 30 min on ice. Cells were washed with 3ml of FACS buffer, centrifuged at 500g for 5 min at 4°C and re-suspended in 200μl of FACS buffer. Cells were analysed using a FACSCantoII flow cytometer and FACSDiva (BD) and FlowJo software (Tree Star). AM were gated as Gr-1^-^F4/80^+^CD11c^hi^CD11b^low^, total lung macrophages were gated as Gr-1^-^F4/80^+^, lung monocytes as Gr-1^-^, CD-11b^+^ and neutrophils as Gr-1^+^ (29). Representative plots shown.

To assess phagocytic activity, the pHRodo *E.coli* phagocytosis kit (Thermo Fisher Scientific, Paisley, UK) was used: pHRodo *E.coli* particles were added to mouse BALF cells, lung homogenates or human MDM and incubated for 2 hr at 37°C with slight agitation, control samples were incubated on ice. Phagocytosis was terminated by transfer of the samples on ice; samples were then processed according to the kit instructions and subjected to flow cytometric analysis.

*Cytokine measurements*

Mouse BALF TNF-α, IL-10 and IL-6 and human TNF-α were measured by ELISA duoset kits R&D Systems (Abingdon, UK). Mouse BALF cytokine profiles were semi-quantitatively assessed using Proteome Profiler™ Mouse XL Cytokine Array Kit membrane based Ab array (R&D systems, Abingdon, UK). Aliquots of BALF samples from 5-6 mice per group were pooled and tested in duplicate. Spot intensity on the array membranes was analysed by densitometry using Image Pro Plus v 5.1 software (Media Cybernetics, Silver Spring, MD, USA).

*Cell viability measurements*

Cell viability was assessed by levels of lactate dehydrogenase (LDH) release in the cell conditioned media using the Cytotoxicity Detection kit (Sigma Aldrich, Dorset, UK).

*Immunofluorescent staining and imaging*

MDM and MitoRed stained MSC co-cultures were seeded onto 8 well culture slides (BD falcon, Oxford, UK) at a 1:20 ratio. After 4 or 24 hr, media was aspirated and cells washed twice with 1x PBS. Cultures were blocked using 1x PBS, 10% normal goat serum (NGS) for 1 hr at room temperature. They were washed twice with 1x PBS and then incubated with primary antibody (human anti-CD45, Abcam, Cambridge, UK) in 1x PBS, 1% NGS at 4°C overnight. Secondary antibody (donkey Alexafluor 405 labelled anti-mouse, Abcam, Cambrige, UK) was then incubated for 1 hr in the same buffer at room temperature and in the dark. Images were obtained by Leica SP5 microscope using the Leica Application Suite AF software and images were analysed using Image J software.

*Measurement of mitochondrial respiration and function*

Macrophage bioenergetics profile was analysed using the Seahorse XF Cell Mito Stress Test Kit (Seahorse Bioscience, MA, USA). MDM were cultured and seeded on 96-well microplates (Seahorse Bioscience, MA, USA) at 20,000 cells/well. MSC were co-cultured at a 1:20 ratio for 24 hr followed by LPS stimulation (10ng/ml) for a further 24 hr. The assay cartridge (XF^e^96, Seahorse Bioscience) was hydrated with the appropriate assay XF calibrant and incubated at 37^o^C without CO_2_ overnight prior to the Mito Stress Test. Cells were then washed in assay media and left at 37^o^C without CO_2_ 1 hr prior to the Mito Stress Test. Oligomycin, FCCP and Rotenone/Antimycin A were diluted to appropriate working concentrations and added to the cartridge followed by the 96-well microplate. The assay was then performed according to the manufacturer’s instructions using the Seahorse XF^e^96 Analyser and analysed using Wave Software.
