# Supplementary figures and images for: Mitochondrial Transfer via Tunneling Nanotubes is an Important Mechanism by Which Mesenchymal Stem Cells Enhance Macrophage Phagocytosis in the In Vitro and In Vivo Models of ARDS
Source: Stem Cells. 2016 Apr 29;34(8):2210–23. doi: 10.1002/stem.2372 (PMC4982045; doi:10.1002/stem.2372)

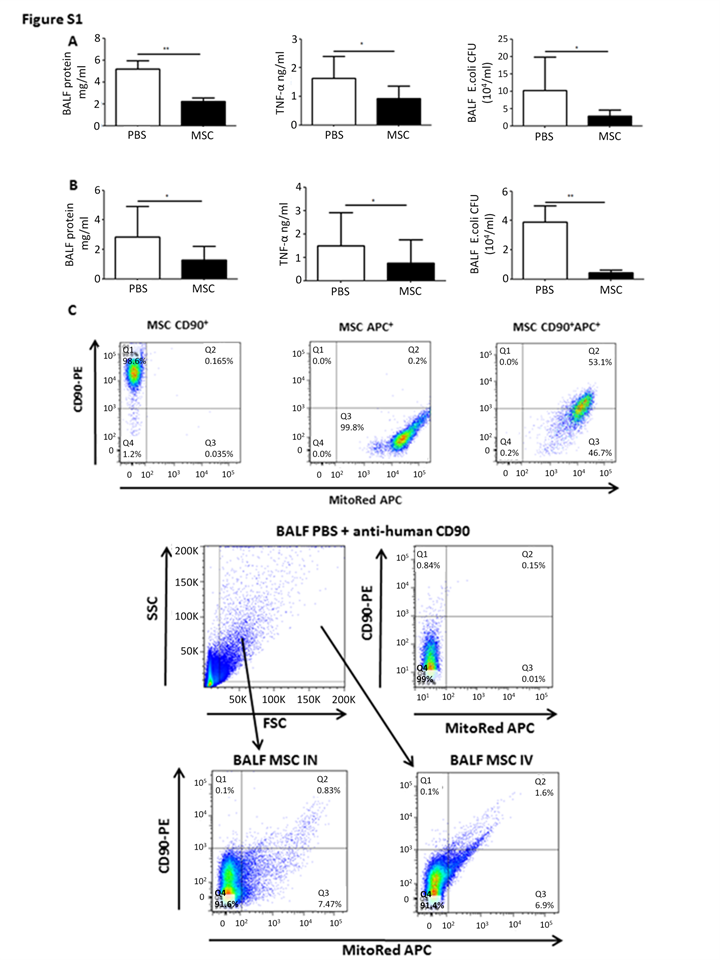

Supplement: Supplementary file 2 — Supporting Information Figure S1 [file STEM-34-2210-s002.tif]

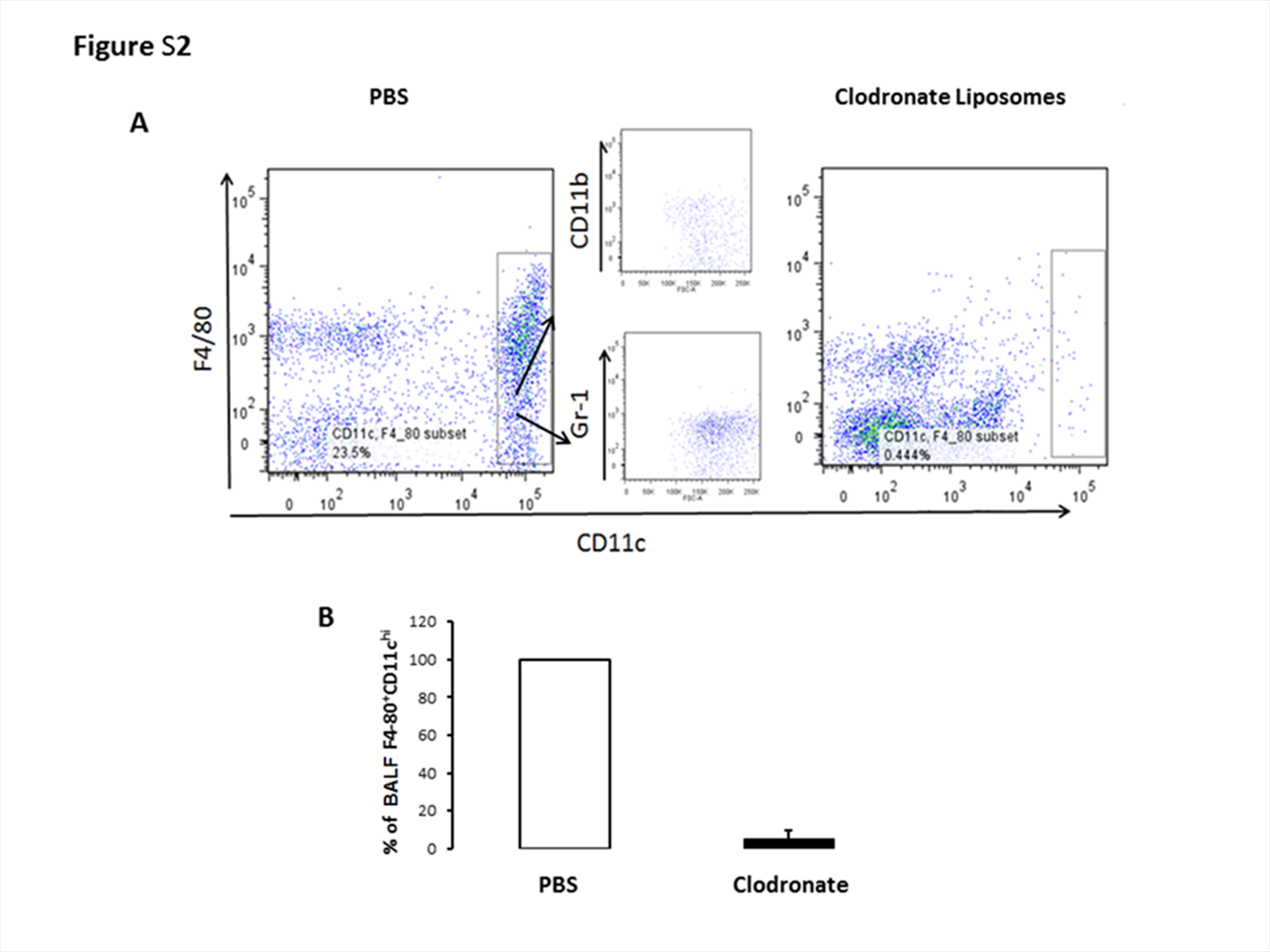

Supplement: Supplementary file 3 — Supporting Information Figure S2 [file STEM-34-2210-s003.tif]

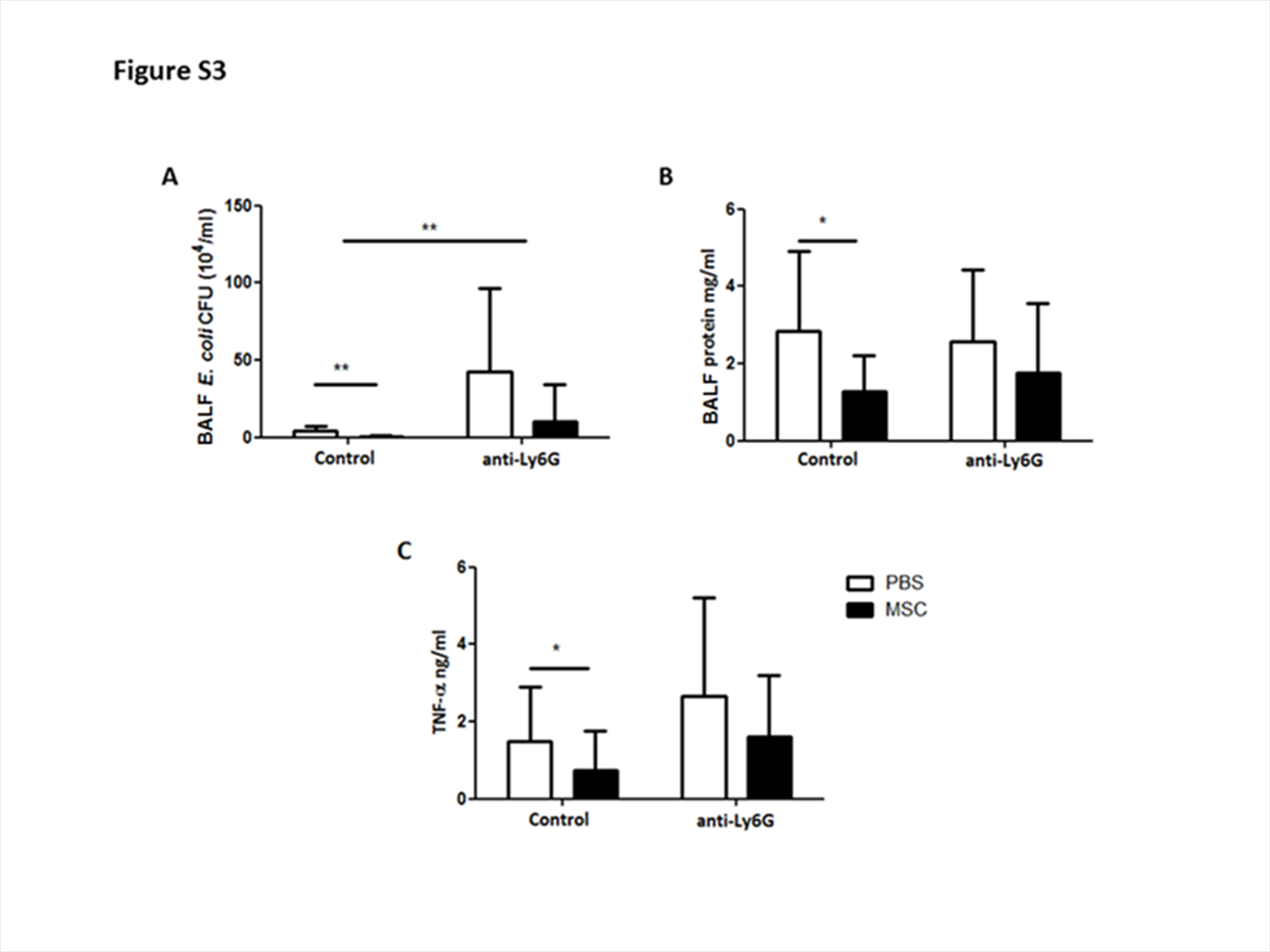

Supplement: Supplementary file 4 — Supporting Information Figure S3 [file STEM-34-2210-s004.tif]

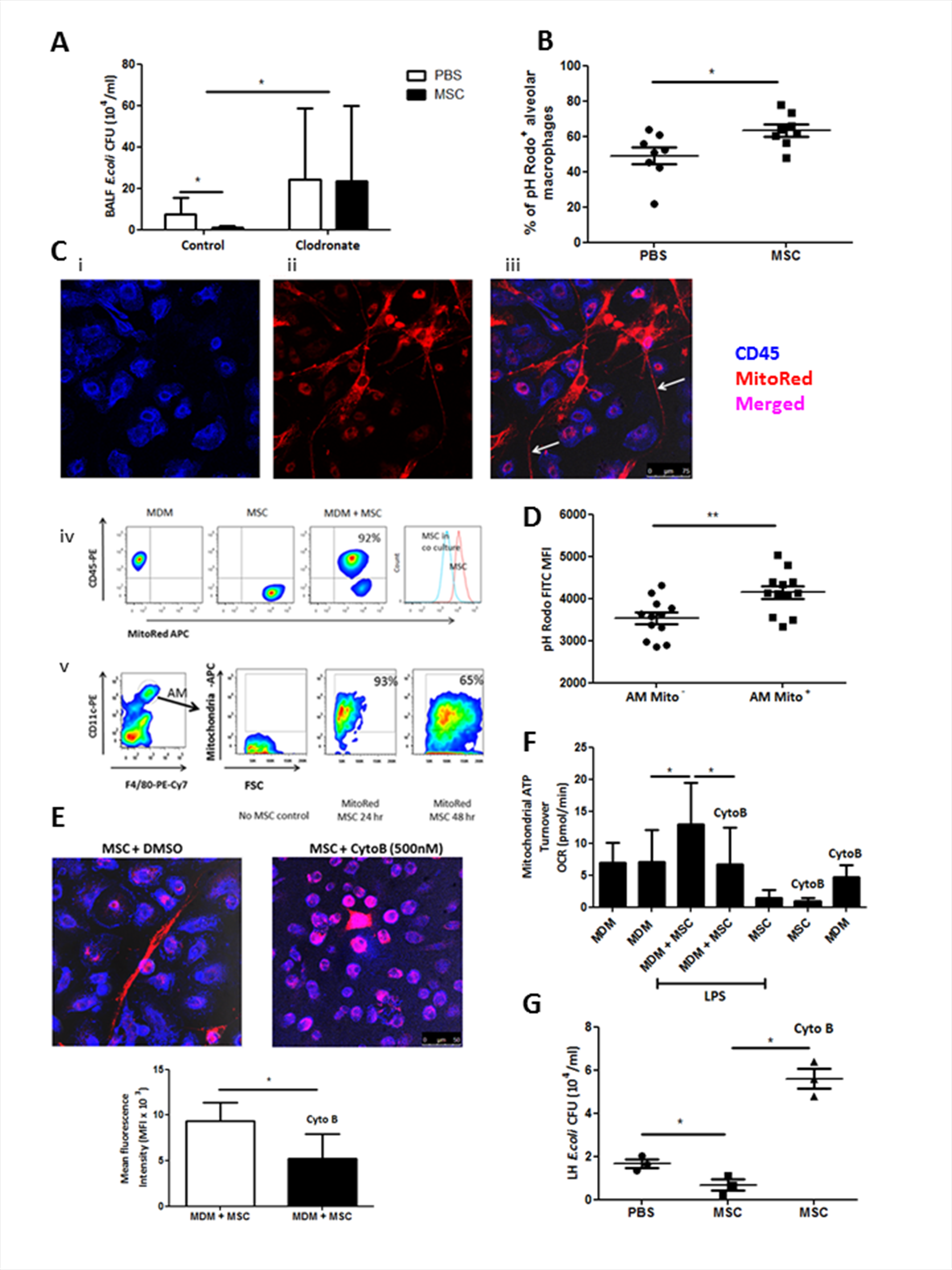

Supplement: Supplementary file 5 — Supporting Information Figure S6 [file STEM-34-2210-s005.tif]
