# Supplementary material for: Mitochondrial Transfer via Tunneling Nanotubes is an Important Mechanism by Which Mesenchymal Stem Cells Enhance Macrophage Phagocytosis in the In Vitro and In Vivo Models of ARDS
Source: Stem Cells. 2016 Apr 29;34(8):2210–23. doi: 10.1002/stem.2372 (PMC4982045; doi:10.1002/stem.2372)
Supplement: Supplementary file 6 — Supporting Information [file STEM-34-2210-s006.docx]

Anti-microbial effect of mesenchymal stromal cells (MSC) in acute respiratory distress syndrome (ARDS) is mediated in part by enhanced alveolar macrophage phagocytosis through TNT-dependent mitochondrial transfer leading to improved macrophage bioenergetics. A) In mouse *E.coli* pneumonia MSC treatment improved bacterial clearance in the lung (p=0.02), alveolar macrophage(AM) depletion by liposomal clodronate significantly increased bacterial burden (p<0.05) and abrogated MSC antimicrobial effect. B) In *E.coli* pneumonia MSC treatment significantly increased the percentage of phagocytic AM compared to PBS suggesting improved AM phagocytosis (p=0.01). C) i-iii) transfer of mitochondria from MSC (MitoRed+) to primary human macrophages (MDM) (CD45+) through TNT-like structures (arrows) (scale bar = 50 μm) iv) Flow cytometry showing more than 90% of CD45+ MDM acquired MSC specific MitoRed fluorescence (APC+), indicating extensive mitochondrial transfer from MSC (4 hr of co-culture) v*) In vivo* 93% and 65% of AM were positive for MitoRed at 24 and 48 hr after MSC administration, respectively. D) AM that had internalised MSC mitochondria (Mito+) showed a significantly higher phagocytic index in comparison to those without (p=0.003). E) Pre-treatment of MSC with Cytochalasin B (500 nM) inhibited TNT formation and partially blocked mitochondrial transfer (scale bar = 50 μm). By flow cytometry pre-treatment of MSC with Cytochalasin B resulted in approximately 50% abrogation in the MitoRed MFI of macrophages (p<0.05). F) Co-culture with untreated but not Cytochalasin B pre-treated MSC significantly enhanced MDM levels of mitochondrial ATP turnover (p<0.05). G) Pre-treatment of MSC with Cytochalasin B abrogated MSC anti-bacterial effect in the *in vivo* E.coli pneumonia model (p<0.05).
